# Antimicrobial Activity and Phytochemical Characterization of *Baccharis concava* Pers., a Native Plant of the Central Chilean Coast

**DOI:** 10.3390/molecules29071654

**Published:** 2024-04-07

**Authors:** Maité Rodríguez-Díaz, Fabián E. Pérez, Paloma M. Manosalva, Juan I. Cerda, Consuelo F. Martínez-Contreras, Aracely Y. Mora, Nicolás A. Villagra, Sergio A. Bucarey, Andrés Barriga, Jorge Escobar, José L. Martínez, Alejandro A. Hidalgo

**Affiliations:** 1 Escuela de Química y Farmacia, Facultad de Medicina, Universidad Andres Bello, Santiago 8370134, Chile; maite.rodriguez@unab.cl (M.R.-D.); fabianperezacevedo@gmail.com (F.E.P.); palomamanosalva@gmail.com (P.M.M.); jcerdarojas15@gmail.com (J.I.C.); consuelom810@gmail.com (C.F.M.-C.); 2 Facultad de Ciencias Químicas y Farmacéuticas, Universidad de Chile, Santiago 8380492, Chile; aracely.mora@ug.uchile.cl; 3 Escuela de Tecnología Médica, Facultad de Salud, Universidad Santo Tomas, Santiago 8370003, Chile; villagra.nicolas@gmail.com; 4 Departamento de Ciencias Biológicas, Facultad de Ciencias Veterinarias y Pecuarias, Universidad de Chile, Santiago 8820808, Chile; sbucarey@uchile.cl; 5 Centro de Estudios Para el Desarrollo de la Química (CEPEDEQ), Facultad de Ciencias Químicas y Farmacéuticas, Universidad de Chile, Santiago 8380492, Chile; anbarr@ciq.uchile.cl; 6 Laboratorio de Química Biológica, Facultad de Ciencias, Pontificia Universidad Católica de Valparaíso, Valparaíso 2340000, Chile; 7 Departamento de Ingeniería Metalúrgica, Facultad de Ingeniería, Universidad de Santiago de Chile, Estación Central, Santiago 9160000, Chile

**Keywords:** *Baccharis concava*, antimicrobial activity, flavonoids, caffeoylquinic acid, phenolic compounds

## Abstract

Few sclerophyllous plants from the central coast of Chile have been systematically studied. This work describes the phytochemical composition and antimicrobial properties of *Baccharis concava* Pers. (sin. *B. macraei*), a shrub found in the first line and near the Pacific coast. *B. concava* has been traditionally used by indigenous inhabitants of today’s central Chile for its medicinal properties. Few reports exist regarding the phytochemistry characterization and biological activities of *B. concava*. A hydroalcoholic extract of *B. concava* was prepared from leaves and small branches. Qualitative phytochemical characterization indicated the presence of alkaloids, steroids, terpenoids, flavonoids, phenolic, and tannin compounds. The antimicrobial activity of this extract was assessed in a panel of microorganisms including Gram-positive bacteria, Gram-negative bacteria, and pathogenic yeasts. The extract displayed an important antimicrobial effect against Gram-positive bacteria, *Candida albicans,* and *Cryptococcus neoformans* but not against Gram-negatives, for which an intact Lipopolysaccharide is apparently the determinant of resistance to *B. concava* extracts. The hydroalcoholic extract was then fractionated through a Sephadex LH-20/methanol–ethyl acetate column. Afterward, the fractions were pooled according to a similar pattern visualized by TLC/UV analysis. Fractions obtained by this criterion were assessed for their antimicrobial activity against *Staphylococcus aureus*. The fraction presenting the most antimicrobial activity was HPLC-ESI-MS/MS, obtaining molecules related to caffeoylquinic acid, dicaffeoylquinic acid, and quercetin, among others. In conclusion, the extracts of *B. concava* showed strong antimicrobial activity, probably due to the presence of metabolites derived from phenolic acids, such as caffeoylquinic acid, and flavonoids, such as quercetin, which in turn could be responsible for helping with wound healing. In addition, the development of antimicrobial therapies based on the molecules found in *B. concava* could help to combat infection caused by pathogenic yeasts and Gram-positive bacteria, without affecting the Gram-negative microbiota.

## 1. Introduction

**Figure 1:**
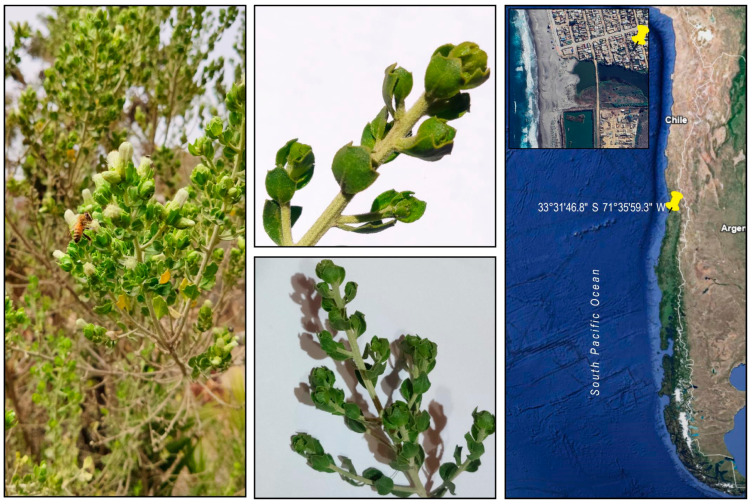
Details of *B. concava* small branches and leaves used for this study. Branches and leaves were collected from a female plant located in a private garden around 400 m from San Sebastian beach and 80 m from the Cartagena estuary, during February (middle summer) 2017. Precise location (33°31′46.8″ S 71°35′59.3″ W) is shown in the right panel, obtained from Google Earth App. By the end of summer (late February and onwards), intense blooming, as seen on the left, massively attracts bees and bumblebees (left panel).

Some native plants of the sclerophyllous type from the central coast of Chile have been studied both for their chemical and biological properties. The present work contributes to these investigations, describing the phytochemical composition and antimicrobial properties of *Baccharis concava* Pers. (sin. *B. macraei*), Asteraceae, a shrub found in the first line and near the Pacific coast between latitudes 29–38° S. See Figure 1 [1]. *B. concava*, locally referred to as *vautro*, has been traditionally used by indigenous inhabitants of today’s central Chile for its anthelmintic, healing, and diuretic properties. A limited number of reports exist regarding the phytochemistry characterization and biological activities of *B. concava*. A hydroalcoholic extract of *B. concava* was prepared from leaves and small branches together. The qualitative phytochemical characterization indicated the presence of alkaloids, steroids, terpenoids, flavonoids, and phenolic and tannin compounds. The antimicrobial activity of this extract was assessed in a panel of microorganisms, including Gram-positive bacteria, Gram-negative bacteria, and pathogenic yeasts. The extract displayed an important antimicrobial effect against Gram-positives, *Candida albicans,* and *Cryptococcus neoformans* but not against Gram-negative bacteria. Therefore, *Salmonella enterica* serovar Typhimurium (*S*. Typhimurium) mutants in enzymes involved in the synthesis of Lipopolysaccharide (LPS) become susceptible to *B*. concava extracts, suggesting that intact LPS is the determinant of resistance to *B. concava* extracts in Gram-negative bacteria. The hydroalcoholic extract was then fractionated through a Sephadex LH-20/methanol–ethyl acetate column. Afterward, the fractions were pooled according to a similar composition that was visualized by TLC/UV analysis. Pooled fractions obtained by this criterion were assessed for their antimicrobial activity against *Staphylococcus aureus*. The fraction presenting the most antimicrobial activity was HPLC-ESI-MS/MS, showing structures derived from phenolic acids and flavonoids such as quercetin, among others.

While systematic studies provide support for popular and ancestral uses of several species of the genus *Baccharis* from South America [2,3,4,5], few studies describe the medicinal properties and phytochemical characteristics of *B. concava* and other species of *Baccharis* in Chile. In 1985, Houghton and Manby found, from a list of 136 species with medicinal properties used by the Mapuche natives in Chile, three species of the *Baccharis* genus: *B. concava* used as a vermifuge; *B. rosmarinifolia*, whose resins have been used for treating rheumatism and also for respiratory and genitourinary infections; and *B*. *sagitallis* for the treatment of fractured limbs [6]. Other species of the *Baccharis* genus such as *B. articulata*, *B. crispa*, and *B. dracunculifolia* have been used for the treatment of ulcers, wounds, and the alleviation of gastrointestinal discomfort and infections, usually used as a decoct of leaves applied on skin or for drinking [7,8]. In 1986, Labbé et al. described ethanolic extracts of *B. linearis*, *B. rhomboidalis,* and *B. solieri*. The partition of these extracts with solvents of higher polarity showed the presence of terpenes, coumarins, and fatty esters of different natures [9]. In 2009, a study reported the antimicrobial and antioxidant activity of aqueous and methanolic extracts of *Baccharis incarum*, which resulted in rich in phenolic acids and flavonoids. The isolated flavones presented antimicrobial activity against methicillin-resistant *S. aureus* and *E. faecalis* [10].

Another Baccharis, specifically *B. tola*, which grows in the Atacama desert in Chile and in the Argentinian Puna, has been extensively studied [11,12]. A diversity of flavonoid and terpenoid compounds were found in the resin rescued from a chloroformic extract, presenting high antioxidant effects.

Preparations of *B. concava* have been used in pre- and post-Columbian traditional medicine to treat wounds, prevent infections, as a diuretic, and as a tonic beverage. To date, there have been few scientific studies describing the medicinal properties and phytochemical characteristics of *B. concava*. In 1989, when preparing dichloromethane extracts of the aerial parts of *B. concava*, Gambaro et al. found derivatives of the clerodane diterpenes, such as hardwickiic acid and hautriwaic acid, and derivatives of bacchasmacranone [13]. Also, using the aerial parts of *B. concava* but performing a hydromethanolic extraction, followed by partitions with petroleum ether, ethyl ether, and ethyl acetate, the flavonoids salvigenin, cirsimaritin, and pectolinarigenin were purified [14]. In 2012, the antimicrobial activity of *B. concava* against *Staphylococcus aureus* (Gram-positive) but not *Escherichia coli* (Gram-negative) was described in essential oils. In addition, the antimicrobial activity of the *Baccharis* genus has been reported in the essential oils of several species [15]. From a total of 102 identified compounds, limonene, miricene, α-pineno, murolene espatulene, δ-cadineno, and lachnophyllum ester were the most abundant, with different proportions depending on the gender of plants [16].

Antibacterial effects have been reported for species of the *Baccharis* genus, including *B. concava*; a wound-healing process is promoted by *B. concava* (according to traditional medicine), and it could be due to antimicrobial effects. Because *B. concava* has not been extensively described in scientific literature, we sought in this study to describe the possible antibacterial effects and phytochemically describe the hydroalcoholic extracts of *B. concava*.

## 2. Results

### 2.1. Alkaloids, Steroids, Terpenes, Flavonoids, and Phenolic Compounds Are Present in the Hydroalcoholic Extract of B. concava

Before testing the biological activity of *B. concava*, the phytochemical composition was qualitatively studied. The extract, obtained in ethanol 70%, had positive results for Dragendorff, Liebermann–Burchard, aluminum chloride, and Ferric chloride, indicating the presence of a detectable amount of alkaloids, steroids, terpenes, flavonoids, and phenolic compounds, as shown in Table 1. In light of the presence of polyphenols and flavonoids in the hydroalcoholic extract of *B*. *concava*, the total concentration of polyphenols and flavonoids was assessed and expressed as equivalent content of gallic acid (GAE) and quercetin (QE), respectively. Total polyphenols in the hydroalcoholic extract of *B*. *concava* extract was equivalent to 799.8 mg GAE/g. Total flavonoids were expressed as quercetin equivalents with a value of 2.27 mg QE/g, while the content of total flavonoids was comparable to other species in the *Baccharis* genus, the content of total polyphenols is remarkably high compared with other *Baccharis* species [17,18,19].

### 2.2. Hydroalcoholic Extract of B. concava Shows Potent Antimicrobial Effects

In a first attempt to find antimicrobial activity, extracts of *B. concava* were directly tested by the agar diffusion test on a lawn of *S. aureus* and *S*. Typhimurium. Because discrete but consistent activity was observed, we decided to test antimicrobial effects on a battery of microorganisms, including Gram-positive, Gram-negative bacteria, and pathogenic yeast by the agar diffusion test. As shown in Table 2, second column, all Gram-positive bacteria were sensitive to the *B. concava* extract. Oppositely, all those Gram-negative-tested (*S*. Typhimurium, *K. pneumoniae,* and *A. baumannii*) were resistant to *B. concava*. The two pathogenic yeasts, *C. albicans* and *C. neoformans*, were also susceptible to the *B. concava* extracts, with *C. neoformans* as the most sensitive microorganism tested, measured as the diameter of inhibition halos. Next, minimal inhibitory concentration was assessed to quantify the concentration capable of inhibiting at least half of the growth (IC_50_) of the tested microorganisms compared with the control condition. As shown in Table 2, third column, all tested microorganisms, except for Gram-negative bacteria, were susceptible to treatment with *B. concava* extract.

Using the same samples of the IC_50_ experiment, treated bacteria with different concentrations were used to prepare base-10 serial dilutions and plated to find the minimal biocidal concentration (MBC), defined as the concentration that kills at least 99.9% of microorganisms (Table 2, fourth column), compared with treatment with the vehicle. As expected, MBCs were higher than MIC-IC_50_, and following the same patterns, *S. aureus* and *S. epidermidis* were among the most susceptible microorganisms when *B. concava* extract was directly diluted in the culture media.

### 2.3. Defective LPS Renders Susceptibility to B. concava Extract in S. Typhimurium

As all tested Gram-negative bacteria were resistant to *B. concava* extract, two plausible hypotheses come to place. First, there are no active compounds against Gram-negative bacteria in *B. concava* extract, or second, Gram-negative bacteria are resistant to active compounds present in *B. concava* extract. Although these hypotheses are not mutually exclusive, the effects of altering the lipopolysaccharide and cell wall stability were tested regarding their effects on susceptibility to *B. concava* extract. Therefore, *S*. Typhimurium mutants in genes *rfaC*, *rfaE*, or *ompA* were exposed to *B. concava* extract. *S*. Typhimurium *rfaC* and *S*. Typhimurium *rfaE* were highly susceptible to *B. concava* extract (see Table 3). This result indicates that affecting the envelope (and therefore the permeability) of *Salmonella* sensitizes the bacteria to the active compound present in the extract; both *rfaC* and *rfaE* genes encode enzymes affecting the synthesis of the LPS core, producing a severely defective LPS molecule. The *ompA* gene encodes for the outer membrane protein OmpA an outer membrane protein, important for anchorage and strength of the cell wall. The mutant in the *ompA* gene of *Salmonella* did not sensitize the bacteria to the extracts.

### 2.4. Column Fractionation of B. concava Extract

**Figure 2:**
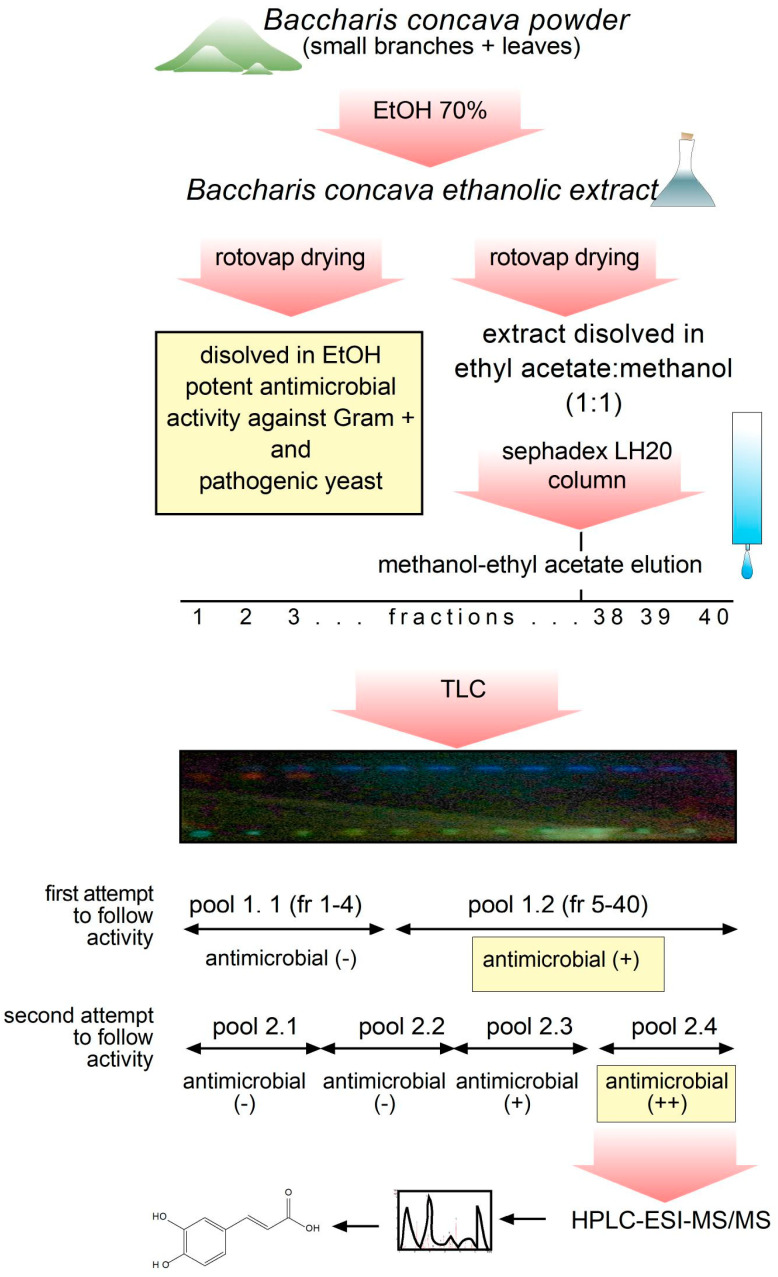
Schematic representation of steps followed from extraction to identification of molecules present in a fraction with antimicrobial activity. A hydroalcoholic extract was obtained from small branches and leaves (all together) and rotovap-dried. After confirming antimicrobial activity in the original hydroalcoholic extract of *B. concava*, dried extract was dissolved in ethyl acetate:methanol (50:50), fractionated in a Sephadex column, analyzed by TLC, and fractions were pooled to test antimicrobial activity. The pool preserving the stronger antimicrobial effects was subjected to HPLC-ESI-MS/MS to identify phenolic compounds.

Sephadex LH-20 column was used to separate 300 mg of dried extract dissolved in 3 mL of methanol/ethyl acetate (1:1). The same solvent was used to elute the extracted compounds. A total of 40 fractions of approximately 5 mL each were collected, resolved by TLC, as described in Section 4, and fractions were pooled according to the presence of similar patterns under UV light. As seen in Figure 2, in a first attempt to follow antimicrobial activity present in the pooled fractions, fractions 1 through 4 and 5 through 40 were pooled to rescue antimicrobials in the bigger pool. Next, in a second attempt, fractions were pooled in lots of 10 consecutive fractions; this time, the fourth pool (pool 2.4 in Figure 2) was the one with the higher antimicrobial activity measured against *S. aureus*. Therefore, pool 2.4 was selected for further chemical composition analysis.

### 2.5. HPLC/Mass Spectrometry Tentative Identification of Phenolic Compounds

**Figure 3:**
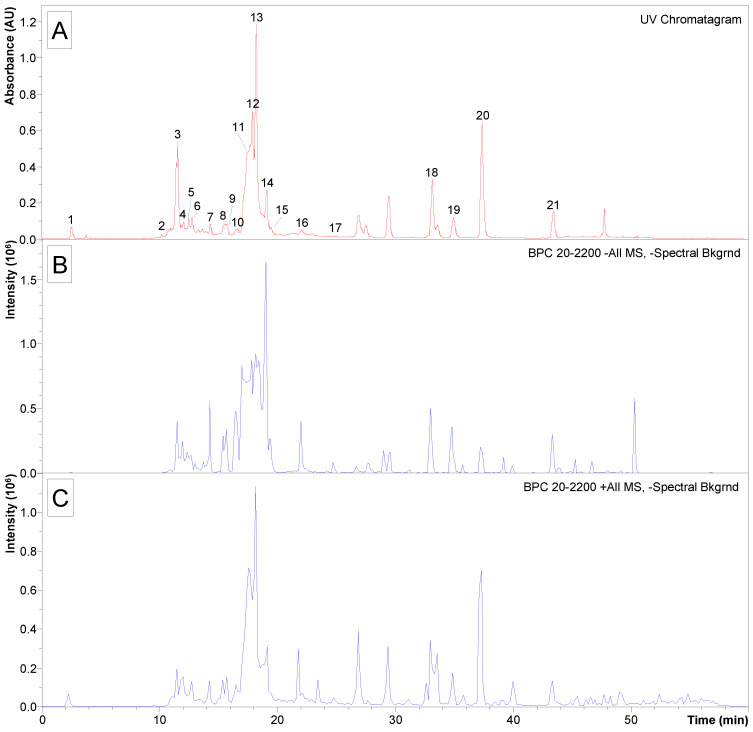
Phenolic profile of the most active fractions of the *Baccharis concava* extract: UV chromatogram (**A**), base peak chromatogram obtained for negative polarity detection (**B**) and base peak chromatogram obtained for positive polarity detection (**C**) of *B. concava* extract with antimicrobial activity. The numbered UV peaks were tentatively identified from the spectrometric data.

Figure 3 shows the UV chromatogram at 254 nm obtained for the *B. concava* extract, pooled fraction 2.4. The identification of the labeled peaks is detailed in Table 4, which contains the precursors observed in negative polarity, as well as their corresponding fragmentations. The fragmentations are arranged by decreasing intensity from left (base peak) to right.

As seen from Table 4, three isomers of caffeoylquinic acid (***m*/*z*** 353, [M-H]^−^) were observed at t_R_ 11.6 min (peak 3), t_R_ 12.2 min (peak 4), and t_R_ 13.3 min (peak 6). Peaks 10–15 were identified as isomers of dicaffeoylquinic acid, as indicated by the observation of signal ***m*/*z*** 515 in negative polarity ([M-H]^−^) and ***m*/*z*** 499 in positive polarity ([M-H_2_O+H]^+^). Peak 17 (t_R_ 24.9 min) would correspond to caffeoylquinic acid-O-hexoside, as suggested by the observation of signal ***m*/*z*** 677 and its fragment ***m*/*z*** 515. Among the flavones, apigenin was identified at t_R_ 33.1 min (peak 18) through signal ***m*/*z*** 269 in negative polarity ([M-H]^−^) and ***m*/*z*** 271 in positive polarity ([M+H]^+^), as well as apigenin-di-C-hexoside (probably vicenin 2) at t_R_ 11.6 min (peak 3) on the basis of signal ***m*/*z*** 593 ([M-H]^−^) and its corresponding fragmentation. Among the flavanols, several quercetin derivatives were identified as peak 5 (t_R_ 12.8 min) and as rutin-O-pentoside based on the signal ***m*/*z*** 741 ([M-H]^−^) and its fragmentation; as peak 7 (t_R_ 14.3 min), which were identified as quercetin-O-rhamnosyl hexoside based on the signal ***m*/*z*** 609 ([M-H]^−^) and ***m*/*z*** 611 ([M+H]^+^) together with their fragmentations; as peak 8 (t_R_ 15. 4 min), identified as quercetin-O-hexoside according to the observation of signal ***m*/*z*** 463 ([M-H]^−^) and ***m*/*z*** 465 ([M+H]^+^); and as peak 9 (t_R_ 15.6 min), which were identified as quercetin-O-glucuronide based on the signals ***m*/*z*** 477 ([M-H]^−^) and ***m*/*z*** 479 ([M+H]^+^) and their corresponding fragmentations. Several kaempferol derivatives were also identified: peak 19 (t_R_ 34.9 min), identified as kaempferol methoxy methyl ether based on the signals ***m*/*z*** 329 ([M-H]^−^) and ***m*/*z*** 331 ([M+H]^+^); peak 20 (t_R_ 37. 3 min), which according to the signal ***m*/*z*** 300 ([M-H]^−^) and its fragmentation would correspond to kaempferol methyl ether (probably kaempferide); and peak 21 (t_R_ 43.4 min), identified as dimethoxy kaempferol according to the signal ***m*/*z*** 313 ([M-H]^−^) and its corresponding fragmentation. Other compounds identified were quinic acid (peak 1), coumarylhexaric acid (peak 2), and caffeoyl feruloylquinic acid (peak 16).

## 3. Discussion

This work describes the antimicrobial activity of a hydroalcoholic extract of *B. concava* against Gram-positive bacteria, *C. albicans*, and *C. neoformans*. Previously, antimicrobial activity was reported against *S. aureus* in essential oil prepared from *B. concava*. In line with our results, the essential oil derived from *B. concava* was inactive against the Gram-negative bacteria *E. coli* [16]. It is possible that the active molecules present in the *B. concava* are inactive against Gram-negative bacteria. However, some *Baccharis* species have presented discrete activity against Gram-negatives; such as the case of *B. revoluta*, although its activity against Gram-positive is higher [20]. Whether the extracts of *B. concava* are inactive against Gram-negative, or Gram-negative are just highly resistant to *B. concava* extracts, was assessed by using *S.* Typhimurium mutants. Increased permeability turned S. Typhimurium susceptible to *B. concava* extracts. Therefore, we speculate that active molecules in the tested extract poorly penetrate the bacterial envelope of Gram-negatives with an intact LPS. Moreover, new evidence indicates that this kind of mutant accumulates oxidative species, which in turn may facilitate killing by antimicrobial agents [21].

Few reports exist about antimycotic effects within the *Baccharis* genus, and none are published for *B. concava*. In this study, we found potent activity against pathogenic yeast *C. albicans* and *C. neoformns*. Previous reports indicate some discrete to poor antifungal activity in some *Baccharis* species; however, some efforts were made to evaluate the synergistic effects of different Baccharis extracts in combination with terbinafine against *Trichophyton rubrum*. Some promising synergistic and additive effects were reported in regard to chemical composition of extracts [22]. Further studies in regard to antimycotic activity will be important in light of emergent yeast and fungi with multiresistant phenotypes [23].

This study describes the phytochemical composition of a polar extract of *B. concava*. Fifteen compounds were identified, and among them, fourteen were described for the first time in this species. These compounds were diterpenoids, flavonoids, and phenolics, which are natural products frequently found in *Baccharis* species. The antimicrobial effects of polar compounds such as phenolic acids, flavonoids, and heteroside derivatives have been reported. An example is chlorogenic acid, a compound found in extracts of *B. concava* whose antimicrobial activity has been reported, probably as a disruptor of bacterial membranes, therefore disrupting cellular homeostasis [24]. Other molecules described in the ethanolic extract of *B. concava* are various caffeic-acid-derivative molecules. Due to the identification of several compounds related to caffeic acid, pure commercial caffeic acid was tested for antimicrobial activity against *S. aureus* using 6 mm filter disks impregnated with 0.3 or 0.5 mg. The effect of pure caffeic acid was discrete, with haloes ranging from 8 to 9 mm, compared with 6 mm in the DMSO control The modest antimicrobial activity observed can be attributed to the low solubility of caffeic acid in water [25]. In the same line, it was reported that caffeic acid improves its antimicrobial activity after encapsulation in cyclodextrin complexes, probably by increasing solubility [26]. It is most likely that the blend of caffeic acid derivatives and other molecules accounts for the potent antimicrobial activity observed in ethanolic *B. concava* extract. Caffeic acid derivatives and other molecules found in this study were previously reported in the related species *B. incarum*, found in Argentina, also showing antimicrobial activity against Gram-positives [10]. Other plants of the Baccharis genus also contain caffeic acid derivatives, including *B. grisebachii*, *B. trimera*, *B. crispa*, and *B. usterii*, found in Argentina and oriental South America [27,28,29]. It is possible to speculate that some caffeic-acid-derivative molecules increase their activity by improving their solubility, stability, and availability. Caffeic acid and its derivative molecules have been isolated from different plant species. The extracts containing such a class of molecule have been extensively tested for their antimicrobial properties, as revised by Khan et al. in 2021 [30]. Moreover, increased antimicrobial activity has been observed for caffeic acid conjugated with other active molecules or vehiculized in nanoparticles [30,31,32,33,34,35,36].

*B. concava* has been used in traditional medicine for wound healing, as a diuretic, and as a tonic in beverages. It is possible that its antimicrobial effects account for its curative effect on wounds. The antimicrobial effects, found in the ethanolic extract, are exclusive to Gram-positive bacteria and pathogenic yeast, with no effect on Gram-negative bacteria. It would be interesting to continue learning about the molecules responsible for the spectrum in the ethanolic extract of *B. concava*, which in the future might allow developing more specific therapies with less unwanted effects on Gram-negative microbiota.

## 4. Materials and Methods

### 4.1. Plant Material

Leaves and small branches of *B. concava* were collected in San Sebastian Beach, V region, Chile (33°31′46.8″ S 71°35′59.3″ W) from a female plant. The collected material was validated by the specialist Scarlett Norambuena and deposited at the Herbarium of Facultad de Ciencias Químicas y Farmacéuticas, Universidad de Chile, under code number SQF#22.889. The leaves and small branches of *B. concava* were dried for 14 days at 21 °C and crushed in a porcelain mortar.

### 4.2. Preparation of Ethanolic Extract

The extracts were prepared by a discontinuous method using ethanol 70% as dissolvent [37]. In brief, 100 g of dry plant powder (leaves and small branches together) was macerated with 1000 mL of ethanol (70% in water) for 3 days at 50 rpm and 30 °C. After 72 h, the extract was filtered and rotovaped from 30 to 70 °C before further drying with a lyophilizer at −50 °C, until the solvents were eliminated. Finally, 42.51 g of dry extract was obtained. The final dried product was stored in an amber bottle at a temperature of 4 °C, according to methodologies reported and standardized in the literature for this type of study [38]. Schematic representation of steps followed from extraction to qualitative identification of molecules is shown in Figure 2. Detailed protocol is described in Supplementary Material.

### 4.3. Phytochemical Characterization

Standard chemical reactions were performed to qualitatively identify the main families of compounds present in the hydroalcoholic extract of *B. concava* [37,39]. The performed tests include Dragendorff for alkaloids, Bornträger for anthraquinones, fluorescence for coumarins, Liebermann–Burchard for steroids and terpenes, aluminum chloride for flavonoids, Keller–Killiani for cardiac glycosides, foam formation for saponins, and Ferric chloride for tannins and phenolic compounds [40]. Details of how to perform these tests have extensively been described in the literature [41,42,43,44,45].

Total polyphenols were measured using the Folin–Ciocalteu method, as described in the literature, based on a calibration curve of GAE in distilled water at concentrations of 50, 100, 250, 350, and 500 μg/mL. Absorbance was measured at 765 nm [46,47]. Total flavonoids were measured in triplicate based on flavonoid–AlCl_3_ complex formation, as described in the literature, based on a calibration curve of QE in ethanol at concentrations of 5, 10, 15, 20, and 25 μg/mL. Absorbance was measured at 420 nm [37,48]. Quantifications were carried out in triplicate.

### 4.4. Antimicrobial Activity Assays by an Agar Diffusion Test

First, 90 mm Petri-like plates were filled with 20 mL of Mueller–Hinton agar. Once the Mueller–Hinton agar solidified, the agar was seeded to form a lawn of bacteria by zigzag streaking a cotton swab soaked in diluted bacteria (McFarland 0,5 in saline serum). Right after seeding the lawn, each plate was 6 mm punched to form 4 holes, 3 peripherics to test 100 μL of the *B. concava* extract (333.33 mg/mL dried extract dissolved in EtOH 70%) and one at the center to test for 100 μL of EtOH vehicle. After incubating for 16 h, the diameter was measured through 3 different sections for each inhibition halo. Independent experiments were performed at least 3 times. To test the susceptibility of yeast, such as *Candida albicans* and *Cryptococcus neoformans*, the assay was carried out as described above, but Mueller–Hinton agar was replaced with Potato–Dextrose agar.

### 4.5. Minimum Inhibitory Concentration (MIC)

Starting with a solution of 166.67 mg/mL of lyophilized extract, base two serial dilutions were prepared in LB-broth (for bacteria) or Potato–Dextrose broth (for yeasts). An amount of 150 μL of each dilution was loaded through the rows of a flat-bottom 96-well plate to finally seed with 50 µL of diluted bacteria or yeast (adjusted to McFarland 0.5 in saline serum). After 16 h of incubation, turbidity of cultures was measured at a longitude of 600 nm to calculate IC_50_, which was consider as the MIC [49]. As a control, bacteria were treated with the same amount of vehicle to look for possible effects on growth.

### 4.6. Minimum Biocidal Concentration (MBC)

From the same set of experiments intended to determine the MIC (or IC_50_), after 16 h of incubation, aliquots of 5 μL from each dilution were seeded on top of an LB agar or Potato–Dextrose agar, incubated for 16 h to finally count the colony forming units (CFU). MBC was estimated as the concentration capable of eliminating 99.9% of microorganisms compared with the control without treatment.

### 4.7. Sephadex Preparatory Column

A 290 mm long × 21 mm wide LH-20 column was selected as the stationary phase to separate 300 mg of dried *B. concava* extract dissolved in 3 mL of methanol/ethyl acetate (1:1). To elute the column, methanol/ethyl acetate (1:1) was used as mobile phase. A total of 40 fractions of approximately 5 mL each were collected, resolved by TLC, and pooled by their patterns, as shown in Figure 2. Finally, antimicrobial activity was determined to continue with further chemical characterization [50].

### 4.8. Thin-Layer Chromatography (TLC)

Characterization of extracts and eluted fractions was carried out by thin-layer chromatography using aluminum oxide (Al_2_O_3_) as the stationary phase and methanol/ethyl acetate (1:1) as the mobile phase. UV (254 nm and 365 nm) and visible longitudes were used to visualize characteristic bands in crude and purified extracts [51].

### 4.9. LC-MS Analysis

The Baccharis extract was examined on an LC-MS system consisting of the HPLC HP 1100 (Agilent Technologies Inc., Santa Clara, CA, USA) coupled to an electrospray ion-trap mass spectrometer Esquire 4000 ESI-IT (Bruker Daltonik GmbH, Bremen, Germany). For the HPLC separation, a Kromasil 100-5C18 250 × 4.6 mm, 5 µm, 100 A column (Eka Chemicals AB, Bohus, Sweden) was used; the column outlet was connected to a split that divided the flow to the UV detector and the mass spectrometer. The analysis was performed at room temperature by the injection of 20 µL of extract at a flow rate of 1.0 mL/min. The mobile phase components were formic acid 0.1% *v*/*v* (component A) and methanol (component B), according to the following elution gradient: 0–5 min, 5% B; 5–7.5 min, 5–20% B; 7.5–20 min, 20–30% B; 20–40 min, 30–40% B; 40–45 min, 40–60% B; 45–50 min, 60–80% B; 50–55 min, 80% B; 55–57.5 min, 80–5% B; and 57.5–60 min, 5% B. The UV detection was performed at 254 nm. The ionization process (nebulization) by electrospray was performed at 3000 V, assisted by nitrogen as nebulizer gas at a pressure of 50 psi and flow rate of 10 L/min, as well as by nitrogen as drying gas at a temperature of 365 °C. Chromatograms and mass spectra were acquired in positive and negative polarity. The trap parameters were set in ion charge control (ICC) using manufacturer default parameters and a maximum accumulation time of 200 ms. Collision-induced dissociation (CID) was performed by collisions with the helium background gas present in the trap. Fragmentation was controlled by SmartFrag. All data obtained were analyzed using DataAnalysis 3.2 (Bruker Daltonik GmbH, Bremen, Germany). The identification of compounds was carried out by comparison of their precursors and corresponding fragmentation patterns with a library developed at the Mass Spectrometry Unit of the Universidad de Chile.

## 5. Conclusions

The hydroalcoholic extracts of *B. concava* show a strong antimicrobial activity, probably due to their composition in phenolic compounds and flavonoids, such as molecules derived from caffeoylquinic acid and quercetin, respectively. Whether a unique compound, a few of them, or a complex combination of secondary metabolites is responsible for the antimicrobial activity needs to be investigated in depth. The development of antimicrobial therapies based on the molecules found in *B. concava* seems attractive and deserves future investigations to confirm antimicrobial activity using in vivo models. Some aspects such as solubility, availability, and stability of both plant extracts and pure metabolites need further investigation to develop efficient antimicrobial therapies. These investigations are important to generate antimicrobial therapies based on new molecules that are effective and specific against pathogens. Of great importance is the antifungal activity of *Baccaris concava* extracts against *C. albicans* and *C. neoformans*. The former yeast is an opportunistic pathogen whose resistance is increasing, while the latter affects immunosuppressed patients and is difficult to treat. Finaly, the effect of the extracts on Gram-positives can be exploited as a specific therapy for this kind of bacteria, without affecting Gram-negative microbiota.

## Figures and Tables

**Table 1:** Qualitative determination of secondary metabolites in an ethanolic extract of *B. concava*.

Assay	Compounds Tested	Positive Results	Result
Dragendorff	alkaloids	red precipitate	+
Bornträger	free Anthraquinones	red color in aqueous phase	−
Fluorescence under UV	coumarins	blue color under UV light	−
Liebermann–Burchard	steroids and terpenes	green–blue or purple–red	+
Aluminium chloride	flavonoids	yellow green fluorescence under UV light	+
Keller–Killiani	cardiac glycosides	greenish blue color	−
Foam formation	saponins	foam production (stands 10 min)	−
Ferric chloride	tannins and phenolic	green or dark blue color	+

Results were either positive (+) or negative (−) according to qualitative visual examination, as the criteriums of the Positive result column were present or absent, respectively.

**Table 2:** Antimicrobial effect of a *Baccharis concava* ethanolic extract.

Microorganism	Inhibition Haloes (mm)	MIC-IC_50_ (mg/mL)	MBC (mg/mL)
*S. epidermidis*	30.67 ± 0.58	6.95 ± 3.01	13.89 ± 6.01
*S. aureus*	20.67 ± 0.58	2.17 ± 0.75	4.34 ± 1.51
*B. subtilis*	14.67 ± 1.16	13.89 ± 6.01	27.78 ± 12.03
*B. cereus*	14.67 ± 1.16	20.83 ± 0	41.67 ± 0
*S. pyogenes*	27.33 ± 0.58	6.95 ± 3.01	13.89 ± 6.01
*E. coli*	6 ± 0	-	-
*S.* Typhimurium	6 ± 0	83.33 ± 0	111.11 ± 48.12
*K. pneumoniae*	6 ± 0	-	-
*A. baumannii*	6 ± 0	-	-
*C. albicans*	14.33 ± 0.58	<27.78 ± 12.03	27.78 ± 12.03
*C. neoformans*	37.00 ± 0	41.67 ± 0	83.33 ± 0

Results are average of three independent experiments ± standard deviation. MIC, minimal inhibitory concentration; MBC, minimal biocidal concentration. Strains were *S. epidermidis* ATCC 12228, *S. aureus* ATCC 25923, *B. subtilis* ATCC 6633, *B. cereus* ATCC 11778, *S. pyogenes* ISP36900, *E. coli* ATCC 25922, *S. enterica* sv. Typhimurium 14028s, *K. pneumoniae* ATCC 13883, *A. baumannii* ATCC 19606, *C. albicans* ATCC 90029, *C. neoformans* GM1. As a control, ampicillin was assessed against *S. aureus*. Five μg/well produced a 22 mm inhibition halo and 0.2 μg/mL reached the IC_50_.

**Table 3:** Mutants of *Salmonella* with defective LPS become susceptible to *B. concava* extract.

*S*. Typhimurium Strains	Function/Structure Affected	Inhibition Halos (mm)
*S*. Typhimurium 14028s WT	Wild-type bacteria	6
*S*. Typhimurium Δ*rfaC*	Synthesis LPS	20
*S*. Typhimurium Δ*rfaE*	Synthesis LPS	22
*S*. Typhimurium Δ*OmpA*	Outer membrane protein/channel	6

Mutants derived from *S*. Typhimurium 14028s (a standard strain) were obtained by facilitated allelic exchange and designed based on mutants obtained through a screening. The *rfaC* gene encodes a heptosyltransferase. The *rfaE* gene encodes a bifunctional enzyme. In both mutants, synthesis of LPS core is affected. The gene *ompA* encodes outer membrane protein A, implicated in transport through membrane and stabilization of cell envelope.

**Table 4:** Tentative identification of compounds present in a *B. concava* extract with antimicrobial activity.

Peak	RT (min)	[M-H]- (*m*/*z*)	Fragments MS2 (*m*/*z*)					Compound
1	2.5	192.1	172.4	126.3	84.4	92.3	110.4	Quinic acid
2	10.9	355.9	190.5	178.5	134.4			Coumaroylhexaric acid
3	11.6	353.2	172.6	178.5	190.5			Caffeoylquinic acid
	11.6	593.7	473.6	503.3	407.8	575.1		Apigenin-di-*C*-hexoside
4	12.2	354.3	190.5	179.0				Caffeoylquinic acid
5	12.8	741.6	300.0	609.2	591.3	475.1	343.1	Rutin-*O*-pentoside
6	13.3	352.2	190.5	178.4				Caffeoylquinic acid
7	14.3	609.5	300.8					Quercetin-*O*-rhamnosyl hexoside
8	15.4	463.6	300.8					Quercetin-*O*-hexoside
9	15.6	477.2	300.9					Quercetin-*O*-glucuronide
10	16.5	515.4	352.9					Dicaffeoylquinic acid
11	17.6	515.1	352.9	190.8	202.8	178.7		Dicaffeoylquinic acid
12	17.8	515.1	352.9					Dicaffeoylquinic acid
13	18.2	515.2	352.9	190.7	178.9			Dicaffeoylquinic acid
14	19.0	515.1	352.9					Dicaffeoylquinic acid
15	19.4	516.6	352.9	202.6	335.1	190.5	172.5	Dicaffeoylquinic acid
16	22.0	529.4	353.1	366.9	190.8	178.7		Caffeoyl-feruloylquinic acid
17	24.9	677.9	515.0					Dicaffeoylquinic acid-*O*-hexoside
18	33.1	269.1	224.7	148.5	200.6			Apigenin
19	34.9	329.1	313.9					Kaempferol methoxy methyl ether
20	37.3	300.0	283.9					Kaempferol methyl ether
21	43.4	313.8	297.2					Kaempferol dimethoxy

## Data Availability

Data are contained within the article and supplementary materials.

## Supplementary Materials

The following supporting information can be downloaded at: https://www.mdpi.com/article/10.3390/molecules29071654/s1.
