# Supplementary material for: Antimicrobial Activity and Phytochemical Characterization of Baccharis concava Pers., a Native Plant of the Central Chilean Coast
Source: Molecules. 2024 Apr 7;29(7):1654. doi: 10.3390/molecules29071654 (PMC11013932; doi:10.3390/molecules29071654)
Supplement: Supplementary file 1 [file molecules-29-01654-s001.zip › molecules-2869768-supplementary.pdf]

Supplementary Material:

Antimicrobial Activity and Phytochemical Characterization of *Baccharis concava* Pers., a Native Plant of the Central Chilean Coast

Maité Rodríguez-Díaz, Fabián E. Pérez, Paloma M. Manosalva, Juan I. Cerda, Consuelo F. Martínez-Contreras, Aracely Y. Mora, Nicolás A. Villagra, Sergio A. Bucarey, Andrés Barriga, Jorge Escobar, José L. Martínez and Alejandro A. Hidalgo

Phytochemical Preliminary Screening

For the detection of different types of secondary metabolites found in the extracts, procedures that involved chemical reactions were carried out, which provided the necessary information to qualitatively explore the presence of alkaloids, flavonoids, saponins, tannins, etc., and they were compared with a positive control. The methods used were as follows:

Alkaloids

Approximately 50 mg of dry extracts were boiled with 6 ml of 2% HCl on a steam bath for 10 min and filtered. The filtrated solution was assayed for the presence of alkaloids, as detailed below.

Dragendorff: A total of 2 ml of the filtrate solution was treated with 4 drops of Dragendorff reagent. A red precipitate indicated the presence of alkaloids [43].

Flavonoids

Approximately 30 mg of the dry extract was dissolved with 8 mL of ethanol and filtered. The filtrate solution was assayed for the presence of flavonoids, as detailed below.

Aluminum chloride test: A few drops of aluminum chloride solution were added to 1 ml of each extract (filtered solution), stirred, and then strips of filter paper were moistened with the mix. The appearance of a yellow spot or yellow–green fluorescence, under ultraviolet light, indicated the presence of flavonoids [44].

Tannins and Phenolic Compounds

Approximately 30 mg of the dry extracts was dissolved in 8 mL of water, heated, and filtered.

Ferric chloride assay: A few drops of 2% $FeCl_3$ were added to 4 ml of filtrated extract. A deep dark green or blue color indicated tannins are present [44].

Steroids and Terpenoids

Libermann–Burchard assay: In a test tube, the extract was dissolved in 2 mL of chloroform. Next, 10 drops of acetic anhydride and 2 drops of concentrated sulfuric acid were added. If the solution turned red, then blue, and finally bluish-green, it indicated the presence of a steroidal nucleus, while the formation of purple or red color indicated the presence of a triterpenoidal nucleus [44].

Saponins

Foam formation assay: Each extract was shaken vigorously with 4 ml of distilled water and allowed to stand for 10 minutes. The stable appearance of foam (standing for at least 10 minutes) indicated the presence of saponins [44].

Cardiac glycosides

Keller–Killiani assay: A portion of dry extract was dissolved in 2 ml of glacial acetic acid, followed by 1 ml of $FeCl_3$ reagent (1 mL of 2% $FeCl_3$ and 99 mL of glacial acetic acid). A few drops of concentrated H_2SO_4 were added to this solution. The presence of blue–green color within a few minutes indicated the presence of a cardiac glycoside deoxy sugar [44].

Coumarins

Fluorescence response assay: The extract was dissolved in 1 ml of ethanol, and a few drops of 1% KOH were added, and then strips of filter paper were moistened with the mix. The formation of blue color under UV indicated the presence of coumarins [44].

Anthraquinones

Borntrager's test: A total of 5 mL of hydroalcoholic extract of the plant was mixed and shaken with 10 mL of chloroform. Then, 5 mL of 10 % ammonium hydroxide solution was added to the filtrate and shaken before the two phases were separated. The development of pink to violet color in the ammoniacal phase (upper part) indicated the presence of free anthraquinones [44].
